# Dealing with lipoedema: women’s experiences of healthcare, self-care, and treatments—a mixed-methods study

**DOI:** 10.1186/s12905-025-03707-1

**Published:** 2025-04-11

**Authors:** Johanna Falck, Annette Nygårdh, Bo Rolander, Lise-Lotte Jonasson, Jan Mårtensson

**Affiliations:** 1 Department of Nursing Science, School of Health and Welfare, Jönköping University, Jönköping, Sweden; 2 Faculty of Caring Science, Work Life and Social Welfare, University of Borås, Borås, Sweden; 3 Academy for Health and Care, Futurum, Jönköping County Council, Jönköping, Sweden; 4 Department of Behavioural Science and Social Work, School of Health Sciences, Jönköping University, Jönköping, Sweden

**Keywords:** Health, Health care quality, Access and evaluation, Lipoedema, Mixed-methods design, Patient experience, Self-care, Surveys and questionnaires, Treatments, Women´s health

## Abstract

**Background:**

Lipoedema is a loose connective tissue disease primarily affecting women characterized by an abnormal build-up of painful fat in the legs and arms. In healthcare, lipoedema is often confused with obesity, and today, diagnostic tools and standardized guidelines for adequate treatments are lacking. Still, research on how affected women manage their health problems and whether they are satisfied with their care remains sparse. Therefore, this study aimed to contribute knowledge on healthcare experiences, and their use and self-reported effects of self-care and treatments among women with lipoedema.

**Methods:**

This national study, with a mixed-methods design, involved 245 women with lipoedema, recruited from all Lipoedema Association groups across Sweden. Data were collected between June and September 2021 through an online survey that included closed- and open-ended questions on self-care, lipoedema treatment, patient satisfaction, and healthcare experiences. Data were analysed using descriptive and inferential statistics, and qualitative reflexive thematic analysis.

**Results:**

The results showed a delay in diagnosis spanning decades, often preceded by numerous healthcare visits. Many women attempted to cope with their health problems using various self-care approaches. However, lipoedema treatments performed by healthcare providers were deemed the most effective. Overall, the women reported significantly low satisfaction with healthcare. The lowest score, 48 points out of 100, was found in the overall impression of offered care, reflecting perceived inefficiency and unmet expectations. Compared to a general Swedish female population, the most significant gaps were found in the dimensions of information and knowledge, and emotional support, 22 and 25 points lower, respectively. The women described their experiences in healthcare as a challenging and isolated journey. Four themes were generated: *A lonely and demanding journey in the healthcare system; An uncertainty of and inconsistency in available healthcare; A burden of being unheard and disrespected in healthcare; and The impact of lack of knowledge in healthcare.*

**Conclusions:**

Seeking care for lipoedema is a long and burdensome journey with limited access to tailored care. Many women make significant efforts to manage their health problems independently. This emphasizes a need for timely lipoedema diagnosis, improved support, and better access to effective treatments.

## Background

Lipoedema is a loose connective (adipose) tissue disease that almost exclusively affects women and manifests as an abnormal accumulation of painful fat, mainly in the buttocks, lower extremities, and arms [1, 2]. Disease onset mainly occurs during periods of hormonal change such as puberty, pregnancy, and menopause. The aetiology of lipoedema is not fully known; however, the female hormone oestrogen, is believed to play a role [3, 4]. Another contributing factor appears to be heredity, as there is often a (female) family history of lipoedema [5–7].

Lipoedema is characterised by nodular and painful fat, easy bruising, numbness, swelling, and heaviness [2] caused by inflammation, fibrosis, and microangiopathy in adipose tissue [8]. Lipoedema mainly affects the lower extremities and sometimes the upper arms. Hands and feet are typically spared [2]. The disease onset primarily occurs during puberty and causes health problems, limitations in daily activities, and lower health-related quality of life, which tends to worsen as the disease progresses [9–11].

Lipoedema is classified into five different types depending on its location in the body [2]. Type 1 affects the buttocks, Type 2 affects the buttocks and thighs, Type 3 extends to the calves, Type 4 involves the arms, and Type 5 affects only the lower legs. Lipoedema is also classified according to disease progression. Stage 1 refers to regular skin but enlarged fat tissue; Stage 2 is uneven skin with indentations in the fat tissue and larger mounds of fat tissue (lipomas) and Stage 3 features large tissue extrusions causing deformations [2]. The clinical presentation of lipoedema varies; however, most women experience gradual progression over time [7, 12]. Diagnosis is based on medical history and a comprehensive physical examination [2, 7].

Currently, there is no cure for lipoedema, and guidelines on how to treat it are generally lacking. However, some approaches and treatments have shown promising results in reducing lipoedema symptoms, improving the quality of life, and preventing disease progression [2]. Offering women with lipoedema educational and psychosocial support to promote self-care - that is, interventions that people can improve upon to maintain health or cope with health problems with or without support from healthcare providers [13, 14] - is essential [2]. Although weight loss has little or no effect on lipoedema, weight management through a healthy diet and physical activity is important for reducing the risk of obesity and obesity-related conditions and improving mobility [2, 15]. Physical exercise should be individualised, and women with severely impaired mobility should be encouraged to seek physiotherapy advice for an individual training plan. Conservative treatment involves individually adapted static compression garments, active pneumatic compression devices, manual lymphatic drainage, and deep tissue therapy to reduce fibrosis, all of which reduce discomfort and relieve aches and pain by providing containment and tissue support [16, 17]. Liposuction is a surgical option that reduces pain, bruising, and swelling and improves mobility by treating adipose tissue enlargement [18–20]. Given the variations in disease progression and clinical presentation, it is essential to regularly assess functioning, limitations in daily activities, and health-related quality of life to address individual needs and optimize treatment strategies [21]. Furthermore, as lipoedema often occurs with multiple and diverse comorbidities and health problems such as obesity, hypothyroidism, migraine, fibromyalgia, allergies, and sleep disorders [10, 22], an interdisciplinary approach should be pursued [23].

An important aspect of care is involving and engaging patients in their care and seeing them as equal partners in planning and implementing care tailored to their needs is a person-centred approach [24, 25], which is closely connected to the quality of care in terms of higher patient satisfaction and better health outcomes [26]. Evaluating patient experiences among women living with lipoedema is crucial as previous research has indicated that women living with this widely unrecognized disease often face several barriers in healthcare [27], being frequently misunderstood and stigmatized by healthcare providers [28–30]. Patient-reported experience measures (PREMs) can be used to evaluate the quality of care from a patient’s perspective. The PREMs gathers valuable information about patients’ experiences and satisfaction with healthcare services and provides insights into how patients perceive and evaluate their care. The most common method for measuring PREMs is through questionnaires designed to capture patients’ perspectives on various aspects of their care, including communication with healthcare providers, coordination of care between providers and services, and general impressions of healthcare [31].

In the last decade, research on conservative and surgical lipoedema treatments aiming to improve the quality of life has slowly increased. Still, evidence of their efficacy remains limited, including lipoedema patients’ experiences of treating lipoedema and their satisfaction with healthcare is sparse [32, 33]. In this study, the overall aim was to investigate experiences of healthcare, self-care, and treatments among women with lipoedema. Specific research questions were: Which experiences do women with lipoedema have of seeking care for lipoedema, and how do they describe these experiences? Which self-care approaches and treatments do women with lipoedema use, and how do they perceive their effects?

## Methods

### Study design

This is a national study with a parallel mixed-methods design. Such design includes at least two parallel strands that address aspects of the same research question, conducted simultaneously but analyzed separately. A parallel mixed-method design is useful for the purpose of complementary, i.e., to learn about multiple aspects of the topic being studied and for validation or triangulation to determine whether various data sets support the overall conclusion [34]. In this study, quantitative data from closed questions and qualitative data from open-ended questions were collected through an online survey conducted between June and September 2021. Quantitative data were collected to more comprehensively measure the use and self-reported effects of self-care, lipoedema treatments, and PREM among women with lipoedema. Qualitative data were collected to gain a deeper insight into women’s experiences when seeking care for lipoedema.

### Measurements

The first part of the survey was developed in collaboration with a woman with lipoedema, a spouse of a woman with lipoedema, and a physician from primary healthcare with clinical expertise in lipoedema. This collaboration aimed to enhance the question’s relevance by capturing diverse perspectives. The survey began with questions regarding sociodemographic (age, educational level, occupation), lipoedema characteristics (time point of disease onset; lipoedema type, stage, and diagnosis), and experiences of self-care and different lipoedema treatments. After that, questions were asked on which type of healthcare settings the women had sought care for lipoedema and the healthcare setting for their most recent lipoedema healthcare visit. Thereafter, a questionnaire was followed, wherein the women could rate on a six-point Likert scale the use of sixteen prelisted self-care strategies and lipoedema treatments. The overall question was ‘*Rate the effect you experienced from this (self-care/treatment)*’, with the answer options: 1 = *I have not used this (self-care/treatment)*, 2 = *Very poor effect*, 3 = *Poor effect*, 4 = *Neither poor nor good effect*, 5 = *Good effect*, and 6 = *Very good effect.* The women could also respond in free text if they had tried treatments other than the pre-listed self-care approaches or lipoedema treatments.

The second part of the survey included a validated questionnaire from The Swedish National Patient Survey (NPS), developed by The Swedish Association of Local Authorities and Regions [35]. The NPS consists of generic PREM questionnaires for inpatient and outpatient settings that aim to capture patients’ experiences of healthcare. The NPS questionnaire used in this study included a basic item pool with 25 generic items and another five items intended for primary care settings. For each item, participants were asked to rate their opinion/attitude on a five-point agreement scale ranging from 1 = *No*,* not at all (strongly disagree)* to 5 = *Yes*,* completely* (strongly agree). Each item also had an answer option of *No opinion/not relevant*. The NPS items covered seven dimensions: *Overall impression*,* Emotional support*,* Participation and involvement*,* Respect and treatment*,* Continuity and coordination*,* Information and knowledge*,* and Availability.* Each dimension was scored from 0 to 100, with a higher score indicating higher patient satisfaction. Permission to use the NPS questionnaire in our lipoedema survey and associated manuals for NPS data analysis was obtained from the Swedish Association of Local Authorities and Regions. Reference data for a general Swedish female population were obtained from the NPS survey for primary care through open access to the Swedish Association of Local Authorities and Regions website [36].

Qualitative data were collected in the survey through self-constructed open-ended questions to explore how participants experienced healthcare in relation to each NPS dimension. The first question was: *‘Describe your experience of the overall process of seeking care for lipoedema’*. Thereafter, seven questions were followed with the same structure addressing the NPS dimensions. The question was: ‘*Describe your experience of (the NPS dimension) in healthcare encounters regarding your lipoedema.*’.

### Data collection procedure

Information about the study, an invitation to participate, an informed consent form, and a link to the survey were sent via email to all members (approximately 700) of all known (*n* = 5) Lipoedema Association groups across Sweden. These groups included women with lipoedema, support members (e.g. family members), and healthcare providers with a particular interest in lipoedema. However, the inclusion criteria were female, aged 18 years or older, and diagnosed with lipoedema or having lipoedema symptoms. The survey distribution (one email followed by two reminders) was conducted in cooperation with the board members of all Lipoedema Association groups. A total of 245 women with lipoedema participated in this survey. The data were collected between June and September 2021. Before full-scale data collection, the survey was tested on 15 women volunteers with lipoedema to identify potential logistical problems, assess the questionnaire’s adequacy, and estimate variability in responses. Ten women provided written feedback on how the questionnaires were carried out, how much time was required to respond to the survey, and suggestions on content and construction, which led to minor revisions for the final survey.

### Statistical analysis

Quantitative data were analysed using the IBM SPSS Statistical Package for Social Sciences version 27.0. Sociodemographic data, lipoedema characteristics, healthcare visits, and effects of self-care and lipoedema treatment were compiled with descriptive statistics. The NPS questionnaire generated data at the ordinal level, which were weighted, calculated, and averaged into dimensional scores according to a standardised NPS manual. Mean differences in the NPS dimensions between participants and the general female Swedish population were analysed with inferential statistics using Student´s *t*-tests [37]. The significance level was set at *p* < 0.05. Cronbach’s alpha was used to calculate item–scale correlations in the NPS dimensions, with results ranging from 0.78 to 0.96.

### Qualitative analysis

Qualitative data (i.e., the answers from the open-ended questions addressing the NPS dimensions) were analyzed using thematic analysis according to Braun and Clarke [38], an approach that was deemed appropriate for this study as it allows for broad flexibility when exploring patterns in qualitative data. The software tool NVivo [39] version 14 was used to systematically handle this large dataset containing 120-word pages, including between 185 and 202 free-text answers to each question. First, the data were read several times to familiarise with the content, including making notes of things of interest and getting ideas that could be of interest to explore further in coding. In the next phase, the data was analyzed as a whole, capturing 20 codes of the essential features. Thereafter, the codes were explored to identify the broader patterns of meaning. This generated seven candidate themes. After identifying overlaps, the candidate themes were combined and further developed, resulting in four refined, defined, and named themes. The process involved recurrently assessing the alignment between the themes and the research question (i.e., the women’s experiences of seeking care for lipoedema) to ensure the findings remained relevant and meaningful. Despite the sequential phases described above, the analysis was a recursive process, with back-and-forth movement between different stages (reviewing themes, returning to coding, and even data familiarization to ensure the analysis remains accurate and meaningful), which is described in the literature as reflexive thematic analysis [38].

### Integration of the quantitative and qualitative inferences

After separate analyses of the quantitative data from the NPS and the qualitative data from the free-text answers, the inferences from the two strands were, following an approach by Tashakkori et al. [34], integrated by interpreting and discussing them in relation to existing literature. Additionally, practical and theoretical implications were drawn from the combined results.

## Results

### Participants’ sociodemographic, lipoedema characteristics, and diagnosis

A total of 245 women with lipoedema responded to the survey (Table 1). The median age was 50–59 years. Most women had an upper secondary or university education, and approximately 70% worked full- or part-time. The most common age range at lipoedema onset was 12–17 years. Over half of the participants had lipoedema stage 3, and the most common lipoedema type (not presented in Table 1) reported among 58.7% of the women was having a combination of lipoedema from the buttocks to the ankles and arms.

**Table 1:** Participants’ sociodemographic, lipoedema characteristics and diagnosis

Variable	Frequency (*n*)	Percentage
**Age (years)**	245	
18–39	16	6.5
40–49	57	23.3
50–59	97	39.6
60–69	45	18.4
70 and older	30	12.2
**Educational level**	245	
Mandatory/High school	64	26.1
Upper secondary/University	181	73.9
**Occupation** ^**1**^	244	
Employed (working full- or part-time)	170	69.7
On sick leave (wholly or partially)	31	13.5
Unemployed	9	3.7
Studying	9	3.7
Retired	48	19.7
Other occupation	9	3.7
**Age at lipoedema onset (years)**	245	
11 or younger	19	7.8
12–17	99	40.4
18–29	51	20.8
30–39	26	10.6
40–49	24	9.8
50 or older	26	10.6
**Lipoedema stage**	245	
Stage 1	23	9.4
Stage 2	81	33.0
Stage 3	133	54.2
**Whether healthcare for lipoedema was sought**	244	
Yes	216	88.5
No	28	11.5
**Whether lipoedema diagnosis was confirmed**	244	
Yes	186	76.2
No	41	16.8
Do not know	17	7.0
**Age at diagnosis (years)**	186	
12–29	3	1.6
30–39	30	16.1
40–49	52	28.0
50–59	65	34.9
60 or older	36	19.4
**Duration of lipoedema diagnosis**	186	
Less than one year	23	12.4
1–2 years	36	19.4
3–5 years	76	40.9
6–9 years	24	12.9
10–19 years	12	6.5
20–29 years	4	2.2
30 years or longer	11	5.9
**Healthcare visits before lipoedema diagnosis**	186	
Diagnosed at first health care visit	30	16.1
1–2	25	13.4
3–6	44	23.7
7–9	23	12.4
10–19	26	14.0
20 visits or more	38	20.4
**Healthcare professional making the diagnosis**	185	
Lymphatic therapist	77	41.6
Physician in primary care	42	22.7
Physician in specialist care	37	20
Nurse	11	5.9
Physiotherapist	10	5.4
Occupational therapist	1	0.5
Other healthcare profession	7	3.8

^1^ 30 participants chose more than one response option here

In this sample, 76.2% of the women had a confirmed lipoedema diagnosis, and among them, one-third had sought care ten times or more for lipoedema symptoms before being diagnosed with lipoedema. Moreover, most women had received a diagnosis of lipoedema within the last five years. It was about as common to have been diagnosed with lipoedema by a physician as it was by a lymphatic therapist.

Among the 41 participants who had not received a lipoedema diagnosis (participants could respond to more than one answer option), the most common answers were *I have not found a healthcare professional with lipoedema competence* (*n* = 26), followed by *I do not know where or whom to turn to* (*n* = 17). Six participants answered not having received a diagnosis because ‘*I have not sought care yet*,*’* and four responded ‘*I do not require a diagnosis’.* Six women answered, ‘*Other reason’*.

A total of 233 women responded to the free text regarding where they had first heard about lipoedema. The most common answers were (merged into groups) *in the media/social media* (43.8%) or *from a friend/colleague/relative* (21.9%). Approximately one-fifth (18.8%) of the women had first heard of lipoedema *from a healthcare provider*. Other responses included *googling/searching on the Internet* (12.9%) and heard of lipoedema *by a personal trainer/masseur* (2.6%).

Most women (61.5%) had primarily sought care for lipoedema in a public healthcare setting. Among participants, most had sought care for lipoedema from a primary care physician (73.5%), and the last healthcare visit for lipoedema had occurred in outpatient healthcare, of which 95% had been in primary care and 5% in specialized patient clinics (i.e., private liposuction clinics).

### The use and self-reported effects of self-care and lipoedema treatments

**Fig. 1:**
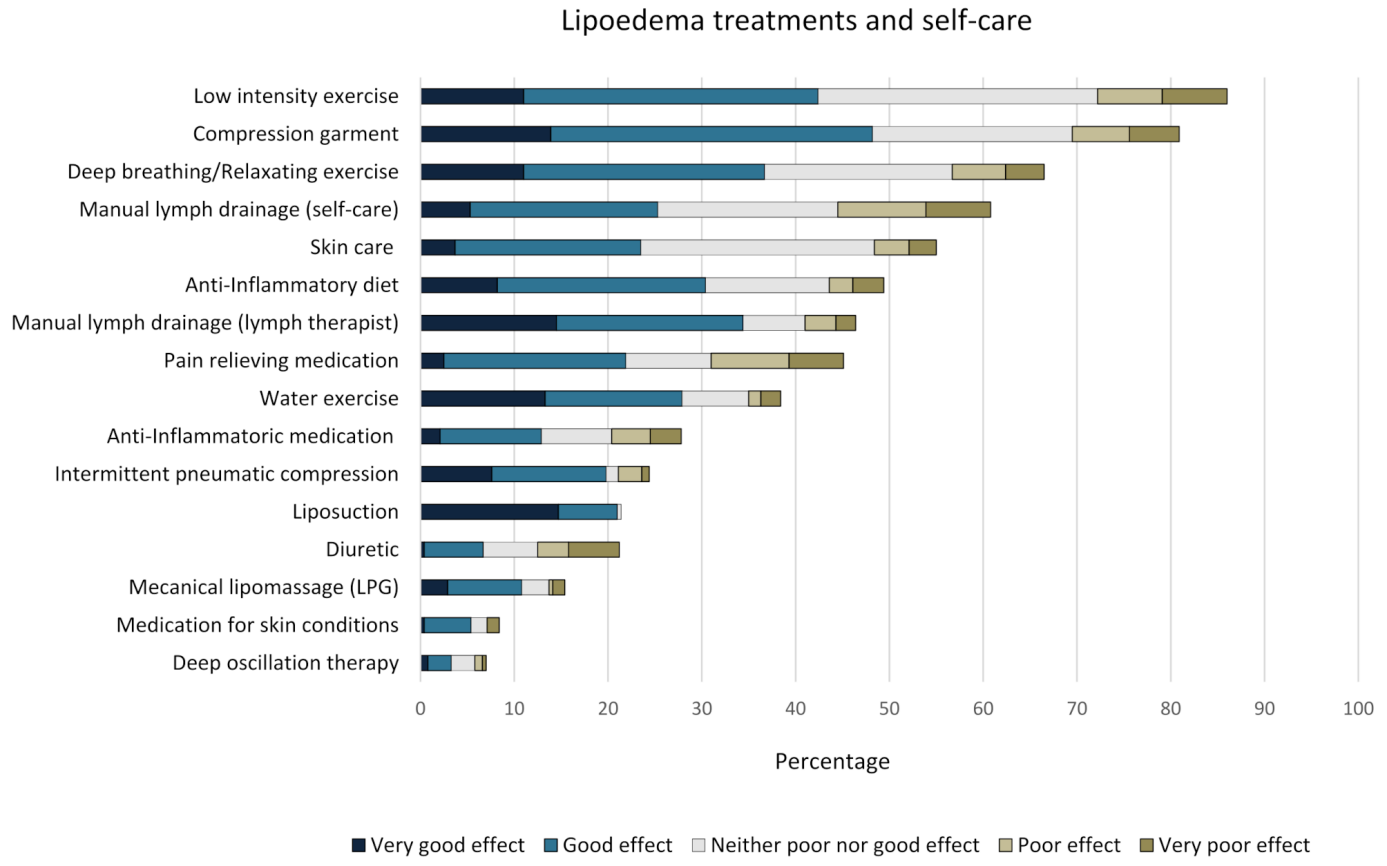
Use and self-reported effect of lipoedema treatments and self-care among participants (*n* = 244)

Most women had tried several strategies to treat their lipoedema symptoms and health problems, and 60% reported using 4–8 lipoedema treatments and self-care approaches. Figure 1 shows the percentages of use and rated effects of different self-care approaches and lipoedema treatments. Most women performed low-intensity exercise, and approximately half rated it as effective (good or very good). Four of five women used compression garments; of those, approximately two-thirds reported good or very good effects. Lymphatic therapy performed by a lymphatic therapist was rated as having a better effect than doing manual lymph drainage as self-care. One-fourth of the women had undergone treatment for lipoedema symptoms with intermittent pneumatic compression devices. Among them, the majority rated the effect as good or very good. In this study sample, 20% had undergone liposuction; all except one participant reported good or very good results. In addition, 42 participants described (in free-text answers) the use of lipoedema treatments or self-care other than those pre-listed. The most common answers were lymphatic massage, a gluten-free and low-carbohydrate diet, and food supplements (magnesium, D-vitamin). Vibration massages with infrared therapy, exercise with a personal trainer, elevating the legs, and using an infrared sauna were also frequently mentioned.

### Experiences of healthcare

**Fig. 2:**
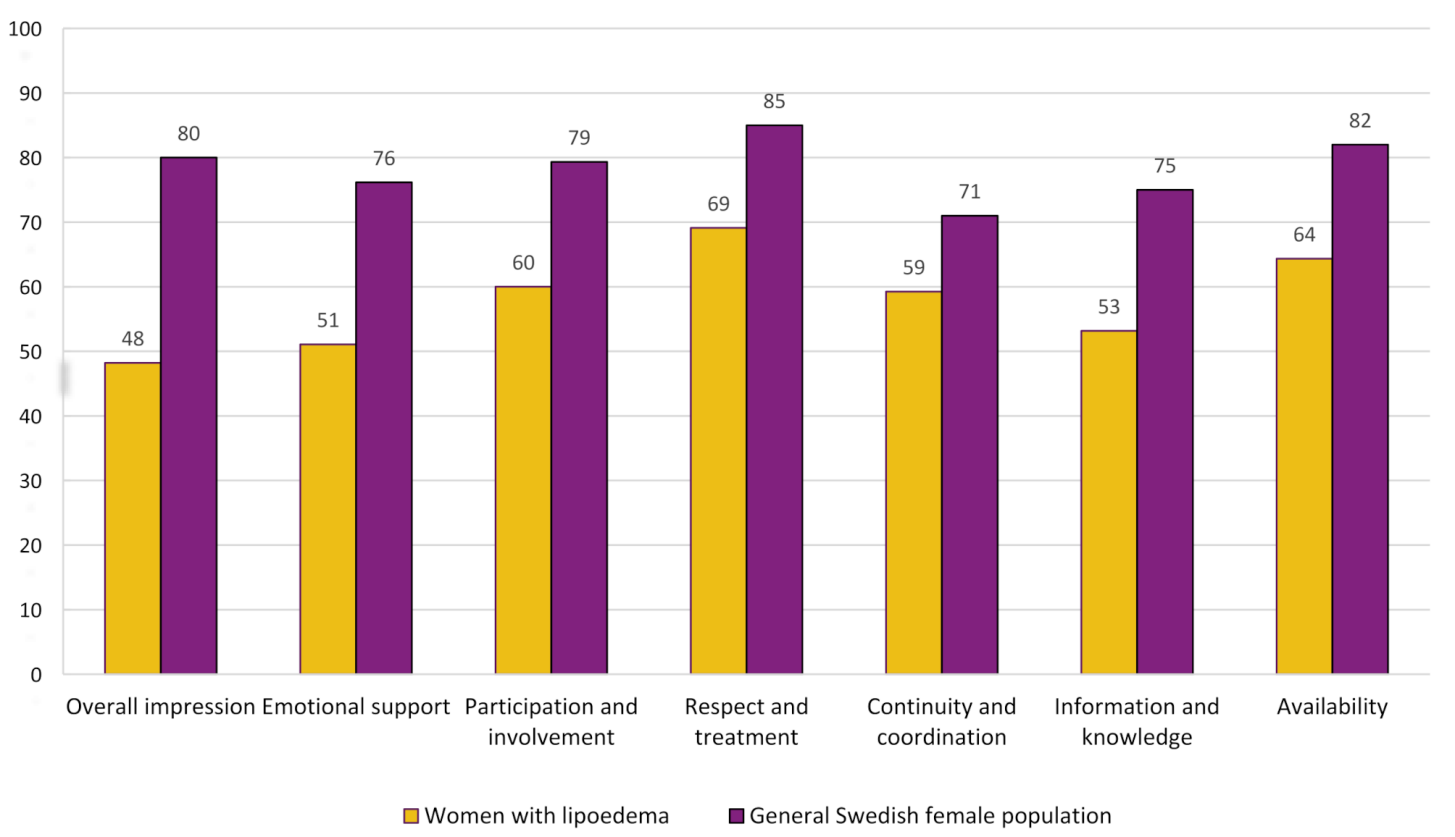
The NPS dimension scores for women who had sought care for lipoedema^1^ (*n* = 209) and a general Swedish female population (*n* = 52,517) in primary. care settings^2^. ^1^In this group, seven of the women did not respond to the NPS questionnaire. ^2^ Significant differences in mean scores between the groups in all seven dimensions = *p* < 0.001

Women with lipoedema scored significantly lower on satisfaction with care in all NPS dimensions than did the general Swedish female population (Fig. 2). The NPS scores ranged from 48 to 69 points for women with lipoedema and from 71 to 85 points for the general Swedish female population. In this study sample, the lowest score (48 points) was found for *Overall impression*, reflecting lower patient satisfaction in terms of expectation of care, perceived efficiency, being cared for, and feeling safe. The dimension of *Emotional support* relates to the experience of healthcare providers being available, responsive, and supportive of patients’ worries, anxiety, pain, or fear. In this dimension, the participants scored 51 points, compared with 76 points in the general female population. Although the highest score among women with lipoedema (69 points) was found in the dimension of *Respect and treatment*, which includes parameters such as compassion, commitment, and care for the patient and individual needs, there was still a difference of 16 points, as this dimension was also found to have the highest score in the general female population (85 points). Moreover, women with lipoedema scored 22 points lower than the general population for *Information and knowledge.* This dimension measures how well the patient feels that healthcare providers can inform and communicate in a well-adapted manner, obtain answers to questions, and receive information about treatments, potential side effects, and warning signs to pay attention to.

The analysis of the qualitative data addressing which experiences women with lipoedema have of seeking care for lipoedema generated four themes: *A lonely and demanding journey in the healthcare system*,* An uncertainty of and inconsistency in available healthcare*,* A burden of being unheard and disrespected in healthcare*, and *The impact of lack of knowledge in care.*

### A lonely and demanding journey in the healthcare system

Overall, the women expressed profound dissatisfaction with lipoedema care. From the perspective of continuity and coordination, the healthcare system was described as challenging, in which no provider took overall responsibility. In general, healthcare providers were found to lack awareness of what to do and how to care for these women. Moreover, the women felt that healthcare providers did not collaborate with other clinics. The women shared experiences of feeling isolated in their efforts to navigate the healthcare system. Without a coordinated care plan, the women described their journey in care as a lonely and demanding struggle with extensive delays, a lack of referrals, and insufficient feedback from healthcare providers. A woman described it as follows:

*‘There is no collaboration or coordination. No referrals. You must search for information about everything and find all the contacts yourself’.*

Participant no 29.

Even if some of the women had met providers who demonstrated a willingness to help, the women were simultaneously informed that the providers were unable to act, as there were no guidelines on which care to offer or whom to refer to. A woman described this as:

*‘My doctor has done all she can—it is not her fault that specialist (lipoedema) clinics do not exist’.*

Participant no 101.

Furthermore, low patient satisfaction regarding participation and involvement in care-related matters was expressed as a sense of loneliness and isolation. The lack of collaboration between healthcare providers left the women feeling abandoned and shut out of their needed care. This lack of coordination was described as being let down:

*‘The Swedish healthcare has let me down…my quality of life just worsens*,* but no one cares’.*

Participant no 98.

### An uncertainty of and inconsistency in available healthcare

The availability of lipoedema care was described as very limited and unequal, and depended mainly on the private economy, meaning if one could pay for care in the private sector. Even though overall, there were high barriers to public care, there were also regional differences, and a few regions offered some support such as referral to a dietician, water exercise, and the prescription of personalised compression garments. In general, the women described lipoedema care as unequal, and the few women with access to lipoedema expertise in public healthcare described it as fortuitous. A woman described it as follows:

*‘It was a lucky coincidence that I came to a doctor who could give me the correct diagnosis after so many years of being misdiagnosed… ’.*

Participant no 26.

Having access to good care was described as an uncertain and often ever-shifting process, as the women suddenly could be left without care when, for example, they or a healthcare provider moved or a healthcare center with lipoedema expertise closed down. This was described by a woman as this:

‘*A physiotherapist in public care had the knowledge*,* but I only was there once because she quit working there. After that*,* I haven’t received any other help’.*

Participant no 92.

### A burden of being unheard and disrespected in healthcare

Regarding experiences from the dimension of respect and treatment in healthcare encounters, the women described a dark picture characterised by a long and often unsuccessful struggle to be heard and acknowledged for their health problems and needs. Their attempts to convince the provider that something was wrong and to be heard were described as useless. The women felt that they were unheard, and that providers instead blamed them for being overweight and questioned them if they were actually telling the truth about their lifestyle behaviour. These experiences were closely related to the dimension of not receiving emotional support for worries and pain, as they often encountered an attitude of ignorance and a lack of interest. Such experiences was described by a woman in the following way:

*‘In every contact with the doctor at the healthcare centre*,* she dismissed my problems with the same comment: “It can’t be like that”. She thought I was making it all up’.*

Participant no 29.

The care providers’ preconception that the women themselves were responsible for their weight gain and health problems was made without being open to whether strategies for losing weight had been attempted before or why they eventually did not work. The women stated that their health problems, medical history, and efforts to manage their health problems were underestimated. A woman described it as follows:

*‘…they see me as a person with very poor self-discipline who gobbles up food and doesn’t exercise because I’m lazy. It is so far from the person I am and has been’.*

Participant no 124.

The experiences of being met disrespectfully and of lacking support affected the women emotionally, causing distress and leaving them feeling disregarded and unsupported. Moreover, the dismissive attitudes of providers contributed to undermining these women’s trust and confidence in healthcare and made them question the value of seeking care in the future. As a woman described:

*‘I have been laughed at in the primary health care centre and told that lipoedema does not exist. I will never go back there again and be humiliated’.*

Participant no 9.

### The impact of lack of knowledge in healthcare

The pervading lack of information and knowledge of lipoedema in public care decreased the women’s opportunities to receive adequate advice on how to manage their health problems and on different options for suitable treatments. A woman described it as follows:

*‘Unfortunately*,* no one in regular healthcare has the knowledge. There are probably exceptions*,* but I have not met them’.*

Participant no 92.

Many women experienced prolonged delays in diagnosis, misdiagnoses, and inadequate assessments of their health conditions. Moreover, the women reported that they did not receive satisfactory answers to questions on how to cope with their health problems or information about different treatment options that could be beneficial. Due to healthcare providers’ limited awareness of lipoedema, these women were forced to seek information about their symptoms independently. When they attempted to share their knowledge about lipoedema during healthcare encounters, it frequently led to miscommunication, creating tension and misunderstanding. Healthcare providers often dismissed the information or questioned whether lipoedema was legitimate, casting doubt on the diagnosis. A woman revealed:

*‘The next doctor I sought help from started googling and stated that lipoedema is a wastebasket diagnosis that I should watch out for. [They] compared it with fibromyalgia’.*

Participant no 70.

However, some providers attempted to find out more by conducting various physical examinations and taking blood tests. However, when a reasonable medical explanation for the symptoms could not be determined, they normalised the women’s problems. As expressed by a woman:

‘*The tests showed that I was perfectly healthy. After some time*,* the pain got even worse*,* and I went to another healthcare centre. Same story’.*

Participant no 98.

For the women, this meant that their care needs were overlooked and disregarded. Therefore, despite disease progression, they continued to face obstacles in accessing appropriate information from healthcare providers regarding how to manage their health issues and the prognosis of their future health.

## Discussion

To the best of our knowledge, this is the first national study involving quantitative and qualitative data on experiences of healthcare, self-care, and treatments among women with lipoedema. The key findings of this study were that women with lipoedema often experienced a delay in lipoedema diagnosis spanning decades, commonly preceded by numerous healthcare visits. Compared to a general female population, women with lipoedema reported significantly lower satisfaction with healthcare in all dimensions. Most of the women treated, with various effects, their lipoedema with different self-care approaches. Even though lipoedema treatments performed in healthcare were perceived as the most effective, they were used less.

The first research question in this study aimed to address the women’s experiences of seeking care for lipoedema. Following the mixed method design, the inferences from the two strands, comprising of data from the NPS questionnaire and free-text answers related to the NPS dimension, are integrated by discussing them in relation to each other and relevant existing literature.

To begin, study participants described the availability of lipoedema care as low, unequal, and unpredictable, depending on either being lucky to have met the ‘right doctor’ or facing a general lack of awareness of lipoedema among providers. Also, the absence of guidelines for lipoedema treatments in public healthcare was pointed out as a barrier to availability. Such obstacles can be discussed in relation to a model of symbolic violence in healthcare, where women with chronic diseases are marginalized through non-recognition (providers deny the condition as a chronic disease), institutionalized medical un-care (no providers take care of the patient), scientific un-care (waiting for more evidence), or con-descension (lack of consensus around the disease) [40]. The model of symbolic violence can contribute to the understanding of why lipoedema remains a “hidden women’s disease” and is not being paid attention to in the broader medical community or society at large. Regarding lipoedema, this means that without a proper acknowledgment of lipoedema as a chronic disease requiring qualified care, many women will continue to be misdiagnosed and left without appropriate medical support.

The dissatisfaction regarding care availability is closely connected to the women’s low patient satisfaction in the NPS dimensions of continuity and coordination in healthcare. Many of the undiagnosed women reported that they lacked guidance on where or from whom to seek help. These results align with how the women described repetitive unsuccessful efforts to navigate the system independently and that they felt left alone. Previous studies have revealed that planning and conducting treatments in interdisciplinary teams with women who suffer from other chronic diseases that have complex etiology and multiple comorbidities like lipoedema has shown promising results in terms of improved functional quality of life and reduced chronic pain [41–43]. Hence, the importance of a holistic approach in addressing the diverse and complex healthcare needs of women with lipoedema cannot be overstated. Collaborative efforts among healthcare providers, including physicians, nurses, lymphatic therapists, physiotherapists, dieticians, psychologists, and other specialists, may provide more integrated healthcare to ensure women receive the best possible care. While the United States, Great Britain, the Netherlands and Germany have provided guidelines that include recommendations for the diagnostic criteria and treatments for lipoedema [2, 17, 21, 44], the absence of published guidelines in Sweden remains an unmet need to improve and expand care for affected women [45, 46]. Furthermore, an implication for clinical practice is the creation of individual care plans for women with lipoedema to address their specific health needs. This may be because each woman receives support and suitable treatment based on her unique medical history and health status. Such an approach could also empower women and help them maintain an active role in their health by providing information and education on optimising their health and well-being [47].

Another key finding of this study was the women’s experiences in healthcare, described as a lonely and exhausting struggle that often extended over many years, colored by disrespect and ignorance from healthcare providers. The low score in the NPS dimensions of respect and treatments, and emotional support corresponded with the theme derived from the qualitative data—the burden of being unheard and disrespected in healthcare. The women’s experiences can be shed in the light of previous research, showing that female sex, large body size, and chronic pain are factors that constitute bias and put women in unfavourable positions in healthcare encounters, wherein healthcare providers tend to ignore or minimize symptom severity or attribute it to a psychological etiology [48, 49]. Gender bias, consolidated by andronormativity in healthcare, is grounded in stereotypes that women are fragile and overemotional, and can affect both care and health outcomes for women, wherein women’s pain and other physical health problems are often attributed to psychosomatic causes [50–52]. Moreover, the perceptions of weight bias among healthcare providers can negatively influence the patient’s experiences and engagement with healthcare services [53]. In this study, several women reported that healthcare providers tended to attribute their health problems to overweight and that they were met with wrong assumptions according to their weight gain. Overall, such biases can constitute barriers for women with chronic conditions when managing health problems and seeking healthcare and have also previously been described among women with lipoedema in diverse international contexts [29, 30]. To provide more equitable care for women with lipoedema, such biases must be recognized and addressed in a healthcare setting. One way to do this is to provide healthcare providers with ongoing training on the impact of care bias [54].

The lack of awareness of lipoedema among healthcare providers was reflected in low scores on the NPS dimension of knowledge and information. The women described this as the main reason for not receiving a diagnosis or adequate information on their health condition. The qualitative data generated a theme- the impact of lack of knowledge in care- pointing out the women’s experiences with healthcare providers failing to explain women’s health problems. It has been previously known that medically unexplained symptoms may strain the relationship between the patient and provider, contributing to mutual feelings of being stuck, lack of trust, and a sense of helplessness [55]. A study claims that for healthcare providers, patients with diffuse symptoms that are difficult to diagnose and treat constitute apparent deviations and are described as difficult and complex [50]. Another study concludes that, although healthcare providers recognize the importance of an adequate explanation or diagnosis of unexplained symptoms and fear neglecting the patient’s symptoms and missing underlying diseases, they face difficulties explaining the nature of the symptoms during their encounters with these patients [56, 57]. Instead, healthcare providers often use different approaches to explain the symptoms to the patient, for example, telling the patient that there is no disease or using metaphors referring to psychological or social problems [57]. In the context of lipoedema, educating healthcare providers about lipoedema is of utmost importance. Moreover, it is essential for providers to listen actively, acknowledge and address the individual needs of women with lipoedema, and collaborate intra-disciplinarily.

The other research question in this study was to examine which self-care approaches and lipoedema treatments the women had used and how they perceived their effects. Important to acknowledge is that many women reported that they had sought care for lipoedema multiple times before being diagnosed, indicating that they had lived a considerably long part of their adult lives without access to well-suited treatments. As such, many women had experiences of using several strategies to treat their health problems independently, and the most common were those that could be performed at home. Although most women performed low-intensity exercise, only approximately half reported positive effects, which aligns with previous research findings [58]. This can be explained by the fact that many women with lipoedema experience daily pain and limitations in physical function [10]. The disproportionate distribution of lipoedema tissue combined with hypermobile joints can negatively affect musculoskeletal health, including orthopaedic conditions such as valgus knee, ankle pronation, gait alterations, arthritis, and knee replacement [2]. Therefore, individually adapted physical therapy regimens, which should be started slowly and advanced based on individual tolerance, are recommended and have shown promising results regarding pain and quality of life in earlier stages [2]. Another key finding in this study was that although the perceived effects of different self-care approaches and treatments varied, treatments performed by healthcare providers, such as manual lymphatic drainage and liposuction, were generally perceived to have the best effect. These findings could be attributed to the fact that co-creating more tailored treatments in healthcare for patients with multi-complex care needs is essential for achieving better health outcomes [59]. Importantly, self-care should not be underestimated as it plays a crucial role in managing lipoedema. Hence, healthcare providers should actively encourage women with lipoedema to adopt or maintain a healthy lifestyle, as it enhances their ability to manage symptoms, improve mobility, and increase quality of life [60]. This can be achieved by offering patient education, individualized care plans, and emotional support, which, in turn, not only contribute to enhancing the women’s overall well-being but also foster a collaborative patient-provider partnership [2, 60]. This study also revealed that more than 80% of the women used compression garments, and the vast majority reported a good or very good effect. It should be acknowledged that this study did not explore the specific effects of different treatments on certain health issues, such as pain, leg heaviness, or mobility. However, similar data from a previous study has shown that women with lipoedema reported that using compression garments was beneficial in reducing pain, supporting tissue, and improving overall comfort [61]. In this study, one-fifth of the women had undergone liposuction, a treatment shown to have the highest proportion of reports of very good effects. Although randomized controlled lipoedema trials on liposuction are currently lacking, a previous study revealed positive results on the quality of life and health status of patients with lipoedema who underwent this treatment [62].

Considering the findings of this study, it is evident that women with lipoedema have challenging journeys in healthcare, characterized by repeated misdiagnoses, disrespectful reception, and low or no access to proper care. This highlights the need for further research and intervention in this area. It is recommended to provide comprehensive education on lipoedema to healthcare providers along with training in addressing biases in care. Furthermore, as lipoedema is a complex disease that affects one’s physical, mental, and social life, the authors of this study suggest developing clinical guidelines to treat and support women with lipoedema. Furthermore, future research should focus on the development and effectiveness of healthcare interventions such as different lipoedema treatments, patient education, and self-management, as well as on studying various dimensions of the psychosocial impact of living with lipoedema. Finally, this study was conducted in the Swedish context, and it should be considered that healthcare systems, cultural norms, and access to care vary significantly across countries. To enhance our understanding of this topic, it is advisable to undertake future investigations encompassing women with lipoedema from diverse populations, embrace cross-cultural comparisons, and foster international collaboration in lipoedema research.

This study has some limitations. Due to non-existent national public authority registers or other Swedish lipoedema data sources, participants were exclusively recruited from Lipoedema Association groups, which may have affected the external validity of these findings. Moreover, information on how many of the 700 members were eligible for inclusion was unavailable, nor could it be confirmed how many of the members actually received the email/study invitation. Therefore, the proportion of confirmed lipoedema diagnoses among the participants in this study is probably not representative of the general lipoedema population. There may also have been selection bias because we had no information about those who did not participate, and one in four of the participants was self-diagnosed. Moreover, this study did not compare the women in groups based on socioeconomic background factors, and the high proportion of educated participants may have skewed the results. All data were self-reported, indicating that there might have been recall bias among participants in questions on lipoedema characteristics and those on their retrospective experiences of the effectiveness of lipoedema treatments. Furthermore, the data collection was conducted through a survey, enabling answers from a large number of participants and enhancing a broad variation of experiences. The prominence of collecting data through written text in qualitative research lies in its time efficiency and practicality for participants [63]. Although the qualitative data was comprehensive, one important consideration is that written text, compared to interviews, is limited by the risk of superficial answers and the lack of contextual depth [63]. However, after evaluating its depth, variation, and contextual details, the data in this study were considered rich and descriptive, containing detailed responses and diverse perspectives. Moreover, a key strength was the mixed-methods approach, where quantitative and qualitative data complemented each other, providing a deeper understanding of the research questions. This type of triangulation, which refers to using multiple methods and data sources [34], contributed to improving the study’s credibility and robustness.

## Conclusions

This study shows that women with lipoedema make great efforts and use several strategies to manage their health problems with little or no support from the healthcare system. The women’s dissatisfaction with care and the low use of lipoedema treatments performed by healthcare providers were primarily described as a result of pervasive low knowledge of lipoedema, repeated misdiagnoses, fat-shaming, and a lack of recommendations for medical treatments. Therefore, there is an urgent need to identify lipoedema in women to enhance timely diagnosis. This should be done by educating healthcare providers regarding lipoedema and offering them continuous training on how to address biases in care. Moreover, to meet the women’s individual needs, provide them with high-quality care, and improve their quality of life, it is critical to develop clinical guidelines for lipoedema, including interdisciplinary collaboration and strategies.

## Data Availability

The datasets generated and analyzed during this study are not publicly available due to ethical restrictions. For further information related to this dataset, please contact the corresponding author.

## Abbreviations

PREMs: Patient Reported Experiences Measurements
NPS: The Swedish National Patient Survey

## References

[1] Ernst AM, Bauer H, Bauer H-C, Steiner M, Malfertheiner A, Lipp A-T. Lipedema research;quo vadis?? J Pers Med. 2023;13(1):98. 10.3390/jpm13010098. 10.3390/jpm13010098 PMC9860653 36675759

[2] Herbst KL, Kahn LA, Iker E, Ehrlich C, Wright T, McHutchison L, et al. Standard of care for lipedema in the united States. Phlebology. 2021;36(10):779–96. 10.1177/02683555211015887. 34049453 10.1177/02683555211015887 PMC8652358

[3] Al-Ghadban S, Teeler ML, Bunnell BA. Estrogen as a contributing factor to the development of lipedema. In: Heshmati HM, editor. Hot topics in endocrinology and metabolism. London: IntechOpen; 2021. 10.5772/intechopen.96402.

[4] Katzer K, Hill JL, McIver KB, Foster MT. Lipedema and the potential role of Estrogen in excessive adipose tissue accumulation. Int J Mol Sci. 2021;22(21):11720. 10.3390/ijms222111720. 34769153 10.3390/ijms222111720 PMC8583809

[5] Grigoriadis D, Sackey E, Riches K, van Zanten M, Brice G, England R, et al. Investigation of clinical characteristics and genome associations in the ‘UK Lipoedema’ cohort. PLoS ONE. 2022;17(10). 10.1371/journal.pone.0274867. 10.1371/journal.pone.0274867 PMC9560129 36227936

[6] Ishaq M, Bandara N, Morgan S, Nowell C, Mehdi AM, Lyu R, et al. Key signaling networks are dysregulated in patients with the adipose tissue disorder, lipedema. Int J Obes. 2022;46(3):502–14. 10.1038/s41366-021-01002-1. 10.1038/s41366-021-01002-1 PMC8873020 34764426

[7] Poojari A, Dev K, Rabiee A, Lipedema. Insights into morphology, pathophysiology, and challenges. Biomedicines. 2022;10(12):3081. 10.3390/biomedicines10123081. 36551837 10.3390/biomedicines10123081 PMC9775665

[8] Al-Ghadban S, Cromer W, Allen M, Ussery C, Badowski M, Harris D, et al. Dilated blood and lymphatic microvessels, angiogenesis, increased macrophages, and adipocyte hypertrophy in lipedema thigh skin and fat tissue. J Obes. 2019;2019:8747461. 10.1155/2019/8747461. 30949365 10.1155/2019/8747461 PMC6425411

[9] Dudek J, Białaszek W, Ostaszewski P. Quality of life in women with lipoedema: a contextual behavioral approach. Qual Life Res. 2016;25(2):401–8. 10.1007/s11136-015-1080-x. 26216585 10.1007/s11136-015-1080-x

[10] Falck J, Rolander B, Nygårdh A, Jonasson L-L, Mårtensson J. Women with lipoedema: a National survey on their health, health-related quality of life, and sense of coherence. BMC Womens Health. 2022;22(1):457. 10.1186/s12905-022-02022-3. 36401222 10.1186/s12905-022-02022-3 PMC9673372

[11] Romeijn JR, de Rooij MJ, Janssen L, Martens H. Exploration of patient characteristics and quality of life in patients with lipoedema using a survey. Dermatol Ther. 2018;8(2):303–11. 10.1007/s13555-018-0241-6. 10.1007/s13555-018-0241-6 PMC6002318 29748843

[12] Langendoen SI, Habbema L, Nijsten TEC, Neumann HAM. Lipoedema: from clinical presentation to therapy. A review of the literature. Br J Dermatol. 2009;161(5):980–6. 10.1111/j.1365-2133.2009.09413.x. 19785610 10.1111/j.1365-2133.2009.09413.x

[13] World Health Organization. Self-care interventions for health. https://www.who.int/health-topics/self-care#tab=tab_1. Accessed 30 Nov 2023.

[14] Socialstyrelsen. Egenvård. https://patientsakerhet.socialstyrelsen.se/risker-och-vardskador/riskomraden/egenvard/. Accessed 30 Nov 2023.

[15] Fetzer A, Wise C. Living with lipoedema: reviewing different self-management techniques. Br J Community Nurs. 2015;20(Sup10):S14–9. 10.12968/bjcn.2015.20.sup10.s14. 10.12968/bjcn.2015.20.Sup10.S14 26418584

[16] Cooper-Stanton G. Adjustable compression devices for chronic oedema and lipoedema: purpose, selection and application. Br J Community Nurs. 2019;24(6):278–82. 10.12968/bjcn.2019.24.6.278. 31166780 10.12968/bjcn.2019.24.6.278

[17] Hardy D, Williams A. Best practice guidelines for the management of lipoedema. Br J Community Nurs. 2017;22(Sup10):S44–8. 10.12968/bjcn.2017.22.sup10.s44. 28961048 10.12968/bjcn.2017.22.Sup10.S44

[18] Baumgartner A, Hueppe M, Meier-Vollrath I, Schmeller W. Improvements in patients with lipedema 4, 8 and 12 years after liposuction. Phlebology. 2021;36(2):152–9. 10.1177/0268355520949775. 32847472 10.1177/0268355520949775

[19] Dadras M, Mallinger PJ, Corterier CC, Theodosiadi S, Ghods M. Liposuction in the treatment of lipedema: A longitudinal study. Arch Plast Surg: APS. 2017;44(4):324–31. 10.5999/aps.2017.44.4.324. 28728329 10.5999/aps.2017.44.4.324 PMC5533060

[20] Wollina U, Heinig B. Treatment of lipedema by low-volume micro-cannular liposuction in tumescent anesthesia: results in 111 patients. Dermatol Ther. 2019;32(2):e12820. 10.1111/dth.12820. 30638291 10.1111/dth.12820

[21] Halk AB, Damstra RJ. First Dutch guidelines on lipedema using the international classification of functioning, disability and health. Phlebology. 2017;32(3):152–9. 10.1177/0268355516639421. 27075680 10.1177/0268355516639421

[22] Ghods M, Georgiou I, Schmidt J, Kruppa P. Disease progression and comorbidities in lipedema patients–a 10-year retrospective analysis. Dermatol Ther. 2020;e14534. 10.1111/dth.14534. 10.1111/dth.14534 33184945

[23] Tuğral A, Bakar Y. An approach to lipedema: a literature review of current knowledge of an underestimated health problem. Eur J Plast Surg. 2019;42(6):549–58. 10.1007/s00238-019-01519-9.

[24] McCormack B, McCance T. Person-Centred Practice in Nursing and Health Care: Theory and Practice. In Riddett J. Person-Centred Practice in Nursing and Health Care: Nursing Management. England; 2017;23(10):15–15.32.

[25] Krist AH, Tong ST, Aycock RA, Longo DR. Engaging patients in Decision-Making and behavior change to promote prevention. Stud Health Technol Inf. 2017;240:284–302. http://www.ncbi.nlm.nih.gov/pmc/articles/pmc6996004/. PMC6996004 28972524

[26] Ekman I, Ebrahimi Z, Olaya Contreras P. Person-centred care: looking back, looking forward. Eur J Cardiovasc Nurs. 2021;20(2):93–5. 10.1093/eurjcn/zvaa025. 33693738 10.1093/eurjcn/zvaa025

[27] Szypłowska M, Gorecka A, Kuś A, Zaremba B, Obel M. Diagnosis and management of lipoedema– a review paper. J Edu Health Sport. 2020;10(9):494–9. 10.12775/JEHS.2020.10.09.059.

[28] Christoffersen V, Tennfjord MK. Younger women with lipedema, their experiences with healthcare providers, and the importance of social support and belonging: A qualitative study. Int J Environ Res Public Health. 2023;20(3):1925. 10.3390/ijerph20031925. 36767290 10.3390/ijerph20031925 PMC9914870

[29] Clarke C, Kirby JN, Smidt T, Best T. Stages of lipoedema: experiences of physical and mental health and health care. Qual Life Res. 2022. 10.1007/s11136-022-03216-w. 35972618 10.1007/s11136-022-03216-w PMC9829602

[30] Melander C, Juuso P, Olsson M. Women’s experiences of living with lipedema. Health Care Women. 2021;1–16. 10.1080/07399332.2021.1932894. 10.1080/07399332.2021.1932894 34252343

[31] Kingsley C, Patel S. Patient-reported outcome measures and patient-reported experience measures. BJA Educ. 2017;17(4):137–44. 10.1093/bjaed/mkw060.

[32] Kamamoto F, Baiocchi JMT, Batista BN, Ribeiro RDA, Modena DAO, Gornati VC. (2025). Lipedema: exploring pathophysiology and treatment strategies - state of the art. J Vasc Bras. 2025;23:e20240025. 10.1590/1677-5449.202400252 10.1590/1677-5449.202400252 PMC11758576 39866170

[33] Lipedema Foundation. FIRST LOOK. Learning by Listening - Early findings from the Lipedema Foundation Registry survey. 2022. https://static1.squarespace.com/static/5775899ac534a5e813c050db/t/6290d2cd923a0156d8fcea1e/1653658317504/LF_First+Look+Registry+Report.pdf Accessed 20 Febr 2025.

[34] Tashakkori A, Johnson B, Teddlie C. Foundations of mixed methods research. Integrating quantitative and qualitative approaches in the social and behavioral sciences. 2nd ed. London: SAGE Publication; 2020.

[35] The Swedish Association of Local Authorities and Regions. Nationell Patientenkät. Rapport Analysuppdrag: Modellutveckling, utvärdering samt tidigare studier och enkäter 2015. https://patientenkat.se/download/18.40c889381840e60521aa1a14/1668006119029/Rapport%20Analysuppdrag_Modellutveckling,%20utv%C3%A4rdering%20samt%20tidigare%20studier%20och%20enk%C3%A4ter_2015.pdf. Accessed 30 Nov 2023.

[36] Swedish Association of Local Authorities and Regions. Nationell patientenkät Primärvård 2021. https://resultat.patientenkat.se/Prim%C3%A4rv%C3%A5rd/2021. Accessed 30 Nov 2023.

[37] Kim HY. Statistical notes for clinical researchers: the independent samples *t*-test. Restor Dent Endod. 2019;44(3):e26. 10.5395/rde.2019.44.e26. 31485422 10.5395/rde.2019.44.e26 PMC6713081

[38] Braun V, Clarke V. Thematic analysis: A practical guide. Los Angeles: SAGE; 2022.

[39] Dhakal K, NVivo. J Med Libr Assoc. 2022;110(2):270–2. 10.5195/jmla.2022.1271. 35440911 10.5195/jmla.2022.1271 PMC9014916

[40] Gimeno Torrent X. The circuit of symbolic violence in chronic fatigue syndrome (CFS)/myalgic encephalomyelitis (ME) (I): A preliminary study. Health Care Women Int. 2022;43(1–3):5–41. 10.1080/07399332.2021.1925900. 34125009 10.1080/07399332.2021.1925900

[41] Allaire C, Williams C, Bodmer-Roy S, Zhu S, Arion K, Ambacher K, et al. Chronic pelvic pain in an interdisciplinary setting: 1-year prospective cohort. Am J Obstet Gynecol. 2018;218(1):114.e1-.e12. 10.1016/j.ajog.2017.10.002. 10.1016/j.ajog.2017.10.002 29031895

[42] Allaire C, Long AJ, Bedaiwy MA, Yong PJ. Interdisciplinary teams in endometriosis care. Semin Reprod Med. 2020;38(2–03):227–34. 10.1055/s-0040-1718943. 33080631 10.1055/s-0040-1718943

[43] Antunes MD, Schmitt ACB, Marques AP. Amigos de fibro (Fibro Friends): development of an educational program for the health promotion of fibromyalgia patients. Prim Health Care Res Dev. 2022;23:e44. 10.1017/s1463423621000773. 35924710 10.1017/S1463423621000773 PMC9381164

[44] Reich-Schupke S, Schmeller W, Brauer WJ, Cornely ME, Faerber G, Ludwig M, et al. S1 guidelines: lipedema. J Dtsch Dermatol Ges. 2017;15(7):758–67. 28677175 10.1111/ddg.13036

[45] Statens beredning för medicinsk och social utvärdering. Lipödem– diagnostik, behandling, upplevelser och erfarenheter. 2021;SBU-rapport nr 327. ISBN 978-91-88437-71-6. https://www.sbu.se/327. Accessed 4 Dec 2023.

[46] Statens beredning för medicinsk och social utvärdering. Prioritering av forskningsfrågor gällande diagnostik, behandling och bemötande av personer med lipödem. 2023;SBU-rapport nr 361. ISBN 978-91-987553-5-0. https://www.sbu.se/361. Accessed 4 Dec 2023.

[47] Coulter A, Entwistle VA, Eccles A, Ryan S, Shepperd S, Perera R. Personalised care planning for adults with chronic or long-term health conditions. Cochrane Database Syst Rev. 2015;2015(3):CD010523. 10.1002/14651858.CD010523.pub2. 25733495 10.1002/14651858.CD010523.pub2 PMC6486144

[48] McManimen S, McClellan D, Stoothoff J, Gleason K, Jason LA. Dismissing chronic illness: A qualitative analysis of negative health care experiences. Health Care Women Int. 2019;40(3):241–58. 10.1080/07399332.2018.1521811. 30829147 10.1080/07399332.2018.1521811 PMC6567989

[49] Wray S, Deery R. The medicalization of body size and women’s healthcare. Health Care Women Int. 2008;29(3):227–43. 10.1080/07399330701738291. 18350426 10.1080/07399330701738291

[50] Claréus B, Renström EA. Physicians’ gender bias in the diagnostic assessment of medically unexplained symptoms and its effect on patient–physician relations. Scand J Psychol. 2019;60(4):338–47. 10.1111/sjop.12545. 31124165 10.1111/sjop.12545 PMC6851885

[51] Heise L, Greene ME, Opper N, Stavropoulou M, Harper C, Nascimento M, et al. Gender inequality and restrictive gender norms: framing the challenges to health. Lancet. 2019;393(10189):2440–54. 10.1016/s0140-6736(19)30652-x. 31155275 10.1016/S0140-6736(19)30652-X

[52] Samulowitz A, Gremyr I, Eriksson E, Hensing G. Brave men and emotional women: A Theory-Guided literature review on gender bias in health care and gendered norms towards patients with chronic pain. Pain Res Manag. 2018;2018:6358624. 10.1155/2018/6358624. 29682130 10.1155/2018/6358624 PMC5845507

[53] Alberga AS, Edache IY, Forhan M, Russell-Mayhew S. Weight bias and health care utilization: a scoping review. Prim Health Care Res Dev. 2019;20:e116. 10.1017/s1463423619000227. 32800008 10.1017/S1463423619000227 PMC6650789

[54] Ramos Salas X, Alberga AS, Cameron E, Estey L, Forhan M, Kirk SFL, Russell-Mayhew S, Sharma AM. Addressing weight bias and discrimination: moving beyond Raising awareness to creating change. Obes Rev. 2017;18:1323–35. 10.1111/obr.12592. 28994243 10.1111/obr.12592

[55] Johansen M-L, Risor MB. What is the problem with medically unexplained symptoms for GPs? A meta-synthesis of qualitative studies. Patient Educ Couns. 2017;100(4):647–54. 10.1016/j.pec.2016.11.015. 27894609 10.1016/j.pec.2016.11.015

[56] Sirri L, Grandi S, Tossani E. Medically unexplained symptoms and general practitioners: a comprehensive survey about their attitudes, experiences and management strategies. Fam Pract. 2017;34(2):201–5. 10.1093/fampra/cmw130. 28122844 10.1093/fampra/cmw130

[57] Olde Hartman TC, Hassink-Franke LJ, Lucassen PL, van Spaendonck KP, van Weel C. Explanation and relations. How do general practitioners deal with patients with persistent medically unexplained symptoms: a focus group study. BMC Fam Pract. 2009;10(1):68. 10.1186/1471-2296-10-68. 19775481 10.1186/1471-2296-10-68 PMC2758831

[58] Donahue PMC, Crescenzi R, Petersen KJ, Garza M, Patel N, Lee C, et al. Physical therapy in women with early stage lipedema: potential impact of multimodal manual therapy, compression, exercise, and education interventions. Lymphat Res Biol. 2022;20(4):382–90. 10.1089/lrb.2021.0039. 34748408 10.1089/lrb.2021.0039 PMC9422785

[59] Kuipers SJ, Cramm JM, Nieboer AP. The importance of patient-centered care and co-creation of care for satisfaction with care and physical and social well-being of patients with multi-morbidity in the primary care setting. BMC Health Serv Res. 2019;19(1):13. 10.1186/s12913-018-3818-y. 30621688 10.1186/s12913-018-3818-y PMC6323728

[60] Williams A, MacEwan I. Accurate diagnosis and self-care support for women with lipoedema. Pract Nurs. 2016;27(7):325–32. 10.12968/pnur.2016.27.7.325.

[61] Paling I, Macintyre L. Survey of lipoedema symptoms and experience with compression garments. Br J Community Nurs. 2020;25(Sup4):S17–22. 10.12968/bjcn.2020.25.sup4.s17. 32271105 10.12968/bjcn.2020.25.Sup4.S17

[62] Schlosshauer T, Heiss C, von Hollen AK, Spennato S, Rieger UM. Liposuction treatment improves disease-specific quality of life in lipoedema patients. Int Wound J. 2021;18(6):923–31. 10.1111/iwj.13608. 33955179 10.1111/iwj.13608 PMC8613387

[63] Polit DF, Beck CT. Resource manual for nursing research: generating and assessing evidence for nursing practice. Eleventh edition. Philadelphia: Wolters Kluwer; 2021.

